# Practice-Pattern Variation in Sedation of Neurotrauma Patients in the Intensive Care Unit: An International Survey

**DOI:** 10.1177/08850666231186563

**Published:** 2023-07-07

**Authors:** Rianne G.F. Dolmans, Brian V. Nahed, Faith C. Robertson, Wilco C. Peul, Eric S. Rosenthal, Marike L.D. Broekman

**Affiliations:** 1 Department of Neurosurgery, 4501 Leiden University Medical Center, Leiden, the Netherlands; 2 Department of Neurology, 2348 Massachusetts General Hospital, Harvard Medical School, Boston, MA, USA; 3 Department of Neurosurgery, 2348 Massachusetts General Hospital, Harvard Medical School, Boston, MA, USA; 4 Department of Neurosurgery, Haaglanden Medical Centre, The Hague, the Netherlands; 5 University Neurosurgical Center Holland, 4501 Leiden University Medical Center, Haaglanden Medical Center and Haga Teaching Hospital, Neurosurgery, Leiden, the Netherlands

**Keywords:** traumatic brain injury, neurotrauma, sedation, neurocritical care, survey

## Abstract

**Background:** Analgo-sedation plays an important role during intensive care management of traumatic brain injury (TBI) patients, however, limited evidence is available to guide practice. We sought to quantify practice-pattern variation in neurotrauma sedation management, surveying an international sample of providers. **Methods:** An electronic survey consisting of 56 questions was distributed internationally to neurocritical care providers utilizing the Research Electronic Data Capture platform. Descriptive statistics were used to quantitatively describe and summarize the responses. **Results:** Ninety-five providers from 37 countries responded. 56.8% were attending physicians with primary medical training most commonly in intensive care medicine (68.4%) and anesthesiology (26.3%). Institutional sedation guidelines for TBI patients were available in 43.2%. Most common sedative agents for induction and maintenance, respectively, were propofol (87.5% and 88.4%), opioids (60.2% and 70.5%), and benzodiazepines (53.4% and 68.4%). Induction and maintenance sedatives, respectively, are mostly chosen according to provider preference (68.2% and 58.9%) rather than institutional guidelines (26.1% and 35.8%). Sedation duration for patients with intracranial hypertension ranged from 24 h to 14 days. Neurological wake-up testing (NWT) was routinely performed in 70.5%. The most common NWT frequency was every 24 h (47.8%), although 20.8% performed NWT at least every 2 h. Richmond Agitation and Sedation Scale targets varied from deep sedation (34.7%) to alert and calm (17.9%). **Conclusions:** Among critically ill TBI patients, sedation management follows provider preference rather than institutional sedation guidelines. Wide practice-pattern variation exists for the type, duration, and target of sedative management and NWT performance. Future comparative effectiveness research investigating these differences may help optimize sedation strategies to promote recovery.

## Introduction

For the 10% to 15% of patients with traumatic brain injury (TBI) who require intensive care unit (ICU) management,^1^ sedatives and analgesics play an important role.^2–5^ First, sedation/analgesia is used for the control of pain, anxiety, and agitation and to enable mechanical ventilation. Second, sedation/analgesia has cerebral protective effects including (1) reduction of the cerebral metabolic rate for oxygen to improve the cerebral tolerance to ischemia and reducing the mismatch between cerebral oxygen demand and supply in conditions of impaired autoregulation. (2) Decreasing the cerebral blood flow in a dose-dependent fashion, leading to a parallel decrease in cerebral blood volume. (3) Consequently, this decrease in cerebral blood volume will produce a reduction of intracranial volume and, therefore, lower intracranial pressure (ICP). (4) Lastly, sedation/analgesia are important for seizure control.^2–5^ In addition, there are also secondary cerebral protective effects of sedation/analgesia such as reducing pain and agitation and improving the tolerance of the endotracheal tube to prevent increases in arterial hypertension and associated elevation in ICP.^4,5^

Given the multiple indications for analgesia and sedation, as well as the limited evidence and guidelines for their use in critically ill TBI patients,^2,3,6^ we hypothesized that significant practice-pattern variation may exist across patients and institutions with a magnitude that may have plausible influences on delirium, mortality, and neurological recovery.^3,7^ As such, we sought to survey providers to quantify practice-pattern variation in analgosedative management of TBI patients requiring ICU management.

## Methods

### Study Design and Survey Development and Distribution

In this survey study, study data were anonymously collected and managed using Research Electronic Data Capture (REDCap) hosted at Massachusetts General Hospital. REDCap is a secure, web-based software platform designed to support data capture for research studies, providing (1) an intuitive interface for validated data capture; (2) audit trails for tracking data manipulation and export procedures; (3) automated export procedures for seamless data downloads to common statistical packages; and (4) procedures for data integration and interoperability with external sources.^8,9^ The authors developed the survey based on the current literature and in consultation with several independent anesthesiologists and neurocritical care physicians. The survey consisted of 56 questions about sedation practice in adult neurotrauma patients in the ICU divided into questions about sedation practice, sedation duration, side effects, and general questions about years of practice, etc. Most questions were multiple choice with the option to specify certain answers. The first two questions of the survey were used to identify neurocritical care workers that participate in the care of TBI patients as well as their sedation management. If participants answered “no” to these questions, the survey was ended automatically to prevent nonneurocritical healthcare workers from completing the survey. The complete survey can be found in the Supplemental Material. Induction sedative agents were defined as “medication used to safely facilitate endotracheal intubation in a manner that minimizes hemodynamic instability and secondary brain injury.” Maintenance sedative agents were defined as “medication as part of the overall management of TBI to permit mechanical ventilation and optimization of intracranial physiology.” Increased ICP was defined as ICP of >20 mm Hg.

The electronic survey was distributed among intensivists, neurosurgeons, neurologists, anesthesiologists as well as ICU physician assistants worldwide via email and Twitter. Participants were emailed directly by the researchers or indirectly through snowballing. Reminder emails to participants were sent twice during the study period. The survey was open from August 3, 2022 until December 5, 2022. This study was done under an institutional review board approved protocol. Informed consent was obtained from all individual participants included in the study.

### Data Collection and Analysis

Descriptive statistics were used to quantitatively describe and summarize the data. Additional stratification was performed for sedation duration in patients with elevated ICP. Two-tailed testing was performed for evaluating each survey question; a *P* value of <.05 was considered significant. Statistical analysis was done using R Studio.

## Results

### Respondents’ Demographics

**Figure 1.**
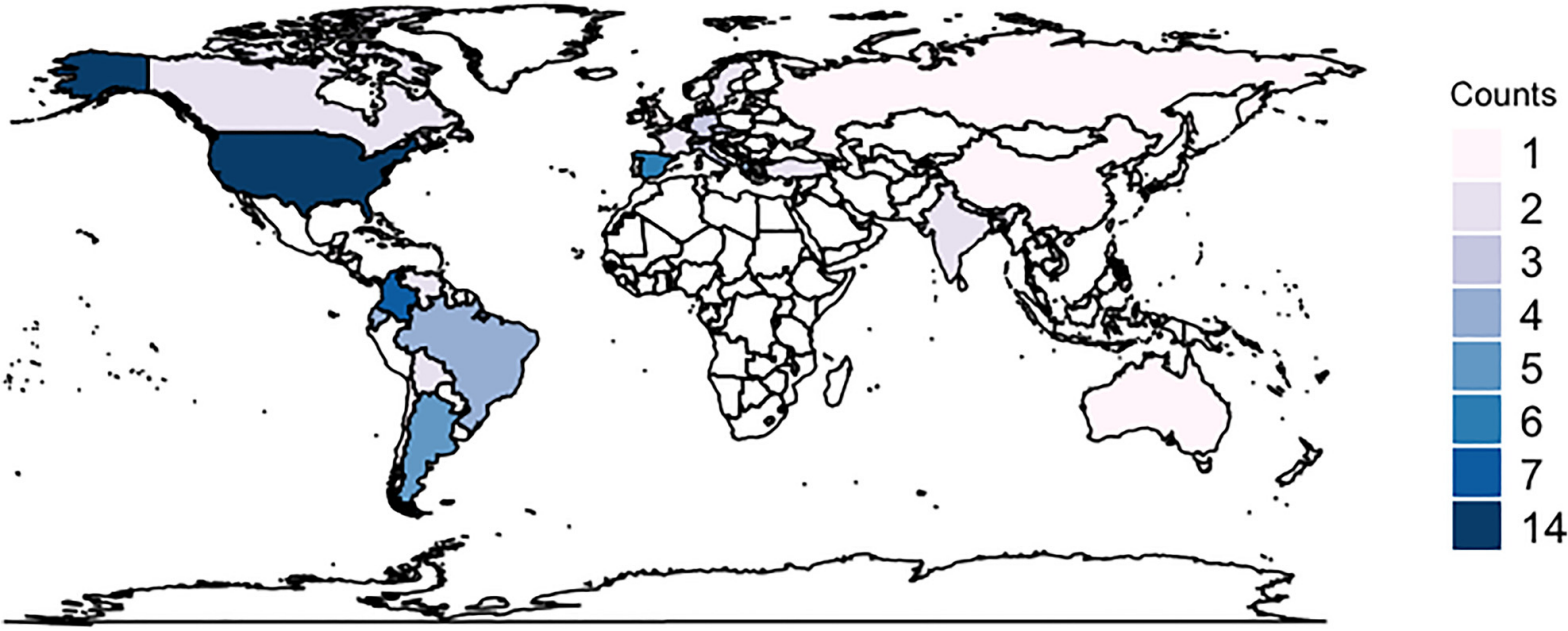
Number of respondents per country.

A total of 95 respondents from 37 countries completed the survey (Figure 1); 56.8% were attending physicians and 22.1% were critical care physician assistants or advanced practice providers (Table 1). Respondents trained in multiple intensive care domains: intensive care medicine (68.4%), anesthesiology (26.3%), neurology (15. 8%), and neurosurgery (13.7%). The median years of experience practicing in the ICU were 12 years (IQR 5.5-20.0). 78.9% of respondents were employed at university hospitals/academic centers, 12.6% at academically affiliated or nonacademic teaching hospitals, 5.3% at community hospitals, and 3.2% in private practices. 31.6% of respondents work in dedicated neuro-ICUs while 28.4% work primarily in a mixed ICU with predominantly neuro-ICU services, surgical-ICU services (13.7%), or medical-ICU services (12.6%). The remaining respondents worked as a consultant in multiple ICUs (5.3%), in a surgical ICU (5.3%), in a medical ICU (2.1%), or other (1.1%).

**Table 1.** Respondents Demographics.

Number of respondents	95
Institution (*n*, %)	
University hospital/academic medical center	75 (78.9)
Subacademic/nonacademic teaching hospital	12 (12.6)
Community hospital	5 (5.3)
Private practice	3 (3.2)
Role (*n*, %)	
Attending physician	54 (56.8)
Critical care physician assistant	21 (22.1)
Fellow	7 (7.4)
Resident	6 (6.3)
Critical care nurse practitioner	3 (3.2)
Other	4 (4.2)
Years of experience (median [IQR])	12.0 [5.5, 20.0]
Medical training (*n*, %)	
Intensive care medicine	65 (68.4)
Anesthesiology	25 (26.3)
Neurology	15 (15.8)
Neurosurgery	13 (13.7)
Emergency medicine	3 (3.2)
Other	4 (4.2)
ICU type (*n*, %)	
Neuro ICU	30 (31.6)
Mixed ICU, predominantly neuro	27 (28.4)
Mixed ICU, predominantly surgical	13 (13.7)
Mixed ICU, predominantly medical	12 (12.6)
Surgical ICU	5 (5.3)
Medical ICU	2 (2.1)
Consultant to multiple ICUs	5 (5.3)
Other	1 (1.1)

Abbreviations: ICU, intensive care unit; IQR, interquartile range.

### Sedation Medication

**Figure 2.**
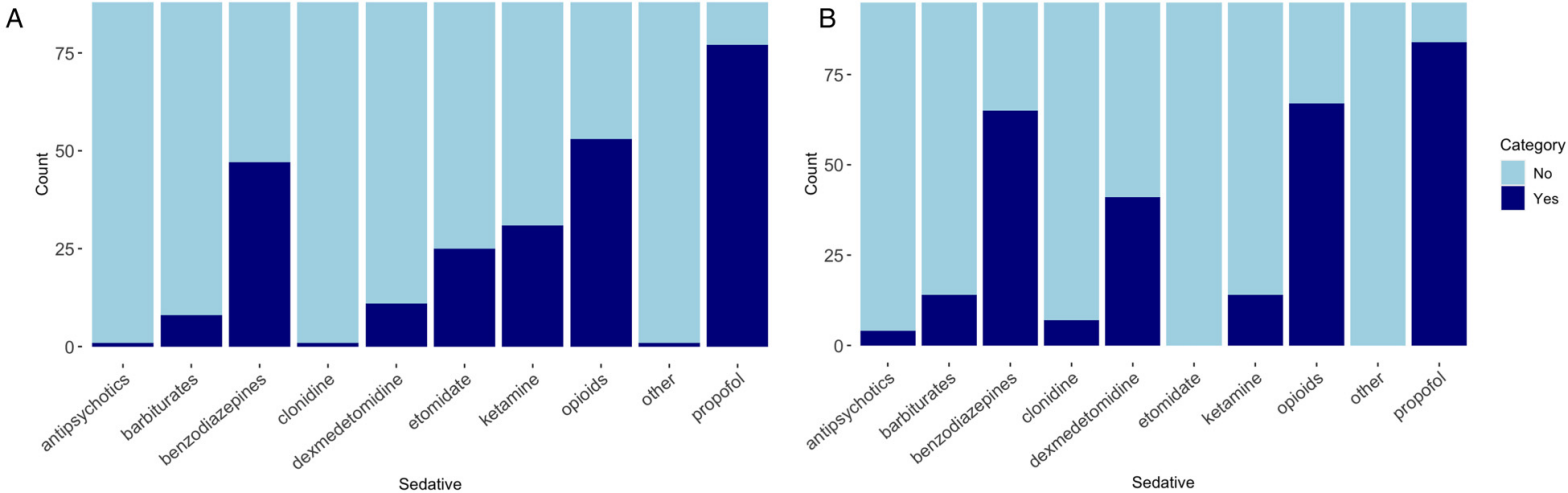
Sedative agents used for (A) induction and (B) maintenance sedation.

**Figure 3.**
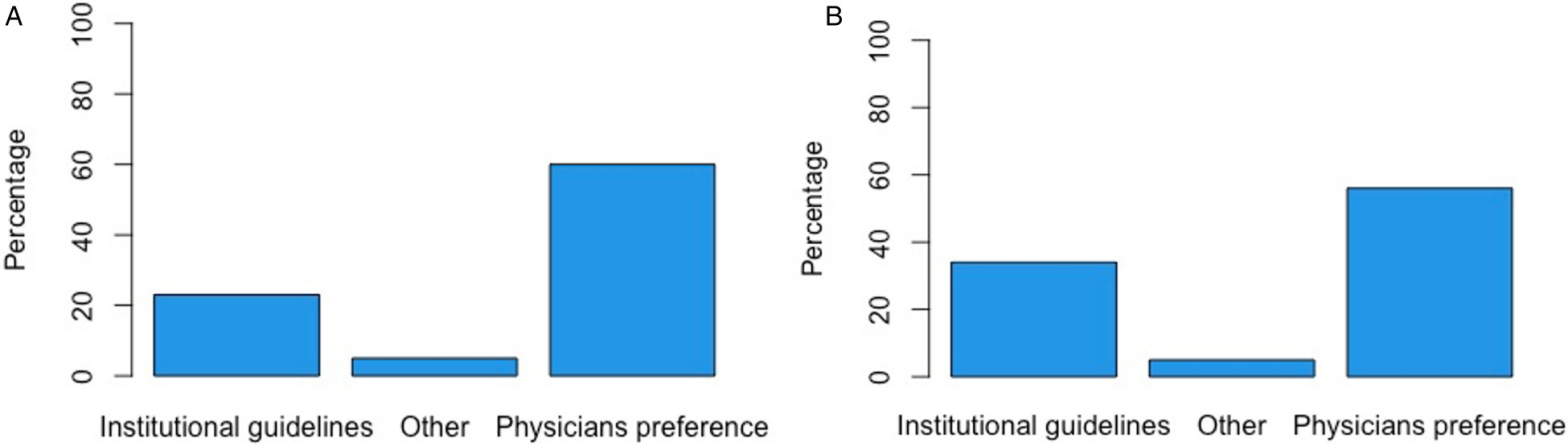
Reason for choice of induction medication (A) and maintenance medication (B).

Institutional guidelines on sedation practice for neurotrauma patients were available in 43.2% of respondents’ institutions (Table 2). The most common sedative agents for induction were propofol (87.5%), opioids (60.2%), benzodiazepines (53.4%), ketamine (35.2%), and etomidate (28.4%) followed by dexmedetomidine (12.5%), barbiturates (9,1%), antipsychotics (1.1%), clonidine (1.1%), or other (1.1%) (Figure 2A). A uniform dose regimen for induction sedative agents in neurotrauma patients was available in 55.7%. The most common reason for using these specific sedative agents for induction was physician preference (68.2%), institutional guidelines (26.1%), or other (5.7%) (Figure 3A).

**Table 2.** Sedation Medication.

Presence of institutional guidelines on sedation (*n*, %)	41 (43.2)
Type of induction medication (*n*, %)	
Propofol	77 (87.5)
Opioids	53 (60.2)
Benzodiazepines	47 (53.4)
Ketamine	31 (35.2)
Etomidate	25 (28.4)
Dexmedetomidine	11 (12.5)
Barbiturates	8 (9.1)
Antipsychotics	1 (1.1)
Clonidine	1 (1.1)
Other	1 (1.1)
Not applicable/not involved during induction phase	7 (7.4)
Reason for induction medication (*n*, %)	
Physician preference	60 (68.2)
Institutional guidelines	23 (26.1)
Other	5 (5.7)
Uniform dose regimen for induction medication (*n*, %)	49 (55.7)
Type of maintenance medication (*n*, %)	
Propofol	84 (88.4)
Opioids	67 (70.5)
Benzodiazepines	65 (68.4)
Dexmedetomidine	41 (43.2)
Ketamine	14 (14.7)
Barbiturates	14 (14.7)
Clonidine	7 (7.4)
Antipsychotics	4 (4.2)
Etomidate	0 (0.0)
Other	0 (0.0)
Reason for maintenance medication (*n*, %)	
Physicians preference	56 (58.9)
Institutional guidelines	34 (35.8)
Other	5 (5.3)
Uniform dose regimen for maintenance medication (*n*, %)	29 (30.5)
Routine use of muscle relaxants (*n*, %)	5 (5.3)
Type of muscle relaxants (*n*, %)	
Rocuronium	3 (60.0)
Succinylcholine	1 (20.0)
Other	1 (20.0)
Routine change of sedation medication after 24 h to 48 h (*n*, %)	30 (31.6)
Sedative agents used after the first 24 h to 48 h (*n*, %)	
Benzodiazepines	17 (56.7)
Opioids	17 (56.7)
Dexmedetomidine	16 (53.3)
Propofol	11 (36.7)
Ketamine	5 (16.7)
Barbiturates	4 (13.3)
Antipsychotics	2 (6.7)
Clonidine	1 (3.3)
Etomidate	0 (0.0)
Other	1 (3.3)

The most common sedative agents used for maintenance were propofol (88.4%), opioids (70.5%), benzodiazepines (68.4%), and dexmedetomidine (43.2%) followed by ketamine (14.7%), barbiturates (14.7%), clonidine (7.4), and antipsychotics (4.2%) (Figure 2B). A uniform dose regimen for maintenance sedative agents in neurotrauma patients was available in 30.5%. The majority of respondents choose maintenance sedative agents based on physicians’ preference (58.9%) rather than institutional guidelines (35.8%) (Figure 3B).

Muscle relaxants were routinely used in TBI patients in the ICU (5.3%); rocuronium (60%) was the most used muscle relaxant followed by succinylcholine (20.%).

31.6% of respondents routinely seek to change sedatives after the initial 24 h to 48 h of ICU admission. The most commonly used sedatives after the initial 24 h to 48 h are benzodiazepines (56.7%) and opioids (56.7%), dexmedetomidine (53.3%), propofol (36.7%), ketamine (16.7%), and barbiturates (13.3%) followed by antipsychotics (6.7%), clonidine (3.3%), or other (3.3).

### Sedation Duration

All respondents (100%) designated that sedation duration in TBI patients is dependent on clinical condition and that there is no fixed duration of sedation (Table 3). The clinical aspects that are evaluated in the duration of sedation are abnormal ICP or multimodality monitoring (94.7%), status epilepticus (65.3%) or refractory seizures (61.1%), Richmond Agitation and Sedation Scale (RASS) score (54.7%), presence of paroxysmal sympathetic hyperactivity (36.8%), or other (7.4%). Sedation duration in patients with increased ICP (>20mm Hg) varied between 24 h and 14 days. For the purpose of this survey, sedation duration was queried in six levels of duration. The sedation duration in TBI patients with status epilepticus, refractory seizures, or paroxysmal sympathetic hyperactivity varied between 24 h and more than 7 days.

**Table 3.** Sedation Duration.

Sedation Duration is Based on (*n*, %)	
Clinical condition	95 (100)
Fixed duration	0 (0.0)
Clinical conditions relevant for sedation duration (*n*, %)	
Abnormal ICP or multimodality monitoring	90 (94.7)
Status epilepticus	62 (65.3)
Refractory seizures	58 (61.1)
RASS level	52 (54.7)
Paroxysmal sympathetic hyperactivity	35 (36.8)
Other	7 (7.4)
Sedation duration in patients with high ICP (*n*, %)	
0 h to 24 h	10 (11.0%)
24 h to 48 h	17 (18.7%)
48 h to 96 h	17 (18.7%)
4 to 7 days	28 (30.8%)
>7 days	4 (4.4)
Until ICP control or definite surgery	15 (16.5%)
Performance of neurological wake-up test (*n*, %)	67 (70.5)
Frequency of neurological wake-up test per hour (*n*, %)	
1	7 (10.4)
2	7 (10.4)
3	1 (1.5)
4	5 (7.5)
6	2 (3.0)
8	6 (9.0)
12	6 (9.0)
24	32 (47.8)
48	1 (1.5)
Time from sedation stop to neurological exam in minutes (mean (SD))	45.2 (38.9)
Reasons for no sedation interruption (*n*, %)	
High ICP	28 (100)
Status epilepticus	20 (71.4)
Refractory seizures	18 (64.3)
Herniation	16 (57.1)
Mass lesion	14 (50.0)
Hypoxia	13 (46.4)
Other	0 (0.0)

Abbreviations: ICP, intracranial pressure; RASS, Richmond Agitation and Sedation Scale.

Neurological wake-up tests (NWTs) to perform a neurologic exam off sedation are done in 70.5%. Most often, this NWT is performed once a day (47.8%) or every 1 h (10.4%) or 2 h (10.4%). On average respondents wait 45.21 (±38.90) minutes to perform a neurological exam off sedation. The reason not to do an NWT for a neurologic exam is high ICP (100%), status epilepticus (71.4%), seizures (64.3%), herniation (57.1%), mass lesion (50.0%), or hypoxia (46.4%).

### Other

**Figure 4.**
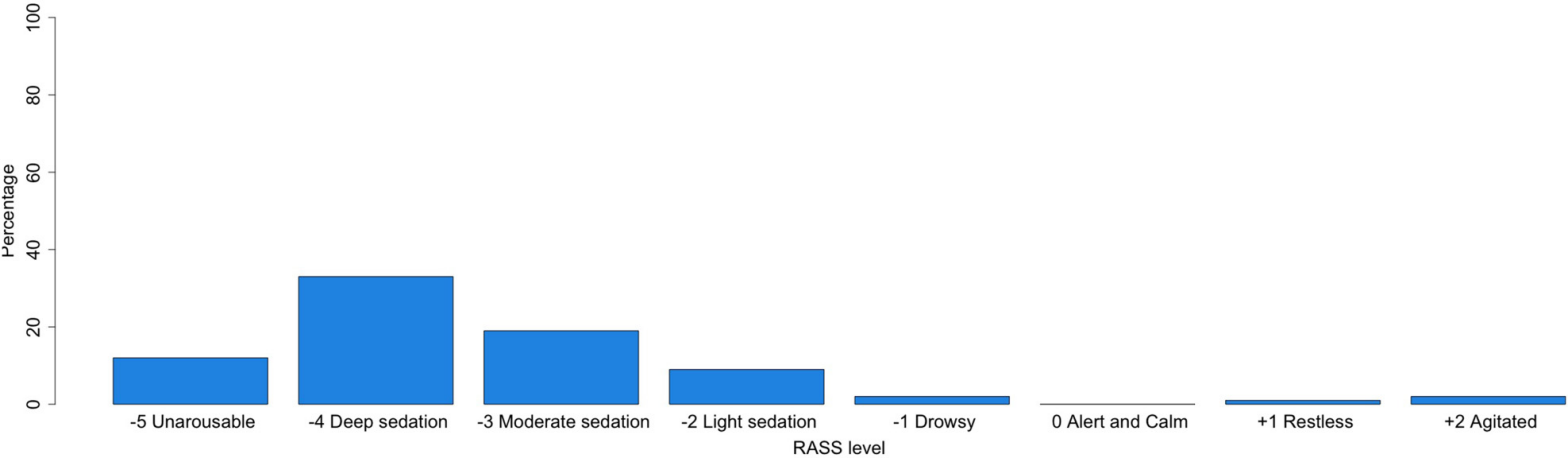
RASS level aimed at TBI patients in the ICU.

Abbreviations: RASS, Richmond Agitation and Sedation Scale; TBI, traumatic brain injury; ICU, intensive care unit.

Initial pharmacotherapy used for elevated ICP is most commonly a combination of osmotherapy and sedatives (66.3%) followed by sedatives only (24.2%) (Table 4). The most common sedative agents used for elevated ICP are propofol (61.6%) and benzodiazepines (24.4%). The most common osmotherapy used for elevated ICP is hypertonic saline (73.6%). The RASS levels targets for critically ill neurotrauma patients varied between deep sedation (level 4; 34.7%) and moderate sedation (level 3; 20.0%) to alert and calm (level 0; 17.9%) or unarousable (level 5; 12.6%) (Figure 4).

**Table 4.** Other.

Initial Pharmacotherapy for Elevated ICP (*n*, %)	
Osmotherapy and sedation	63 (66.3)
Sedation	23 (24.2)
Osmotherapy	9 (9.5)
Osmotherapy for elevated ICP (*n*, %)	
Hypertonic saline	53 (73.6)
Mannitol	19 (26.4)
Sedative agents for elevated ICP (*n*, %)	
Propofol	53 (61.6)
Benzodiazepines	21 (24.4)
Barbiturates	6 (7.0)
Opioids	4 (4.7)
Dexmedetomidine	1 (1.2)
Ketamine	1 (1.2)
RASS level (*n*, %)	
+4 Combative	0 (0.0)
+3 Very agitated	0 (0.0)
+2 Agitated	2 (2.1)
+1 Restless	1 (1.1)
0 Alert and calm	17 (17.9)
−1 Drowsy	2 (2.1)
−2 Light sedation	9 (9.5)
−3 Moderate sedation	19 (20.0)
−4 Deep sedation	33 (34.7)
−5 Unarousable	12 (12.6)
Early tracheostomy to minimize sedation (*n*, %)	43 (45.3)
Side effects (% and SD)	
Respiratory suppression	56.3 (23.9)
Propofol infusion syndrome	18.6 (21.1)
Delirium	51.7 (20.5)
Adrenal suppression	20.8 (20.1)
Hemodynamic instability	58.4 (17.6)

Abbreviations: ICP, intracranial pressure; RASS, Richmond Agitation and Sedation Scale; SD, standard deviation.

45.3% of respondents indicated a preference to perform an early tracheostomy to minimize sedation in neurotrauma patients.

The most common treatment-related side effects of sedation reported for TBI patients by respondents were: hemodynamic instability (58.4%), respiratory suppression (56.3%), delirium (51.7%), adrenal suppression (20.8%), and propofol infusion syndrome (18.6%).

## Discussion

In this international survey of sedation practices for critically ill TBI patients, practice-pattern variations were evident for choice and duration of sedatives, performance and frequency of NWTs, and target depth of sedation. Institutional sedation guidelines were reported by less than half of respondents; most often decisions regarding sedation were made based on physician preference.

### Choice of Sedative Agents

Reasons for this practice-pattern variation may relate to the lack of definitive evidence for an optimal strategy across the diverse range of TBI patients. Several studies have investigated sedative agents in TBI patients in the ICU in relation to cerebral physiology and patient outcome.^2,10–16^ Nevertheless, there is limited evidence on the best choice of sedative agents with each sedative agent having specific advantages and disadvantages. Propofol was the most commonly used sedative agent by respondents in this survey, and sedative agents are often compared with propofol to assess their potential benefits.^5,17–20^ While high-dose propofol may result in significant morbidity and disrupt cerebral autoregulation,^6,16,21^ studies comparing benzodiazepines and propofol have failed to demonstrate a difference in patient outcome.^5,12,17,20^ Midazolam can be a risk factor for ICU delirium, which itself is independently associated with poor outcomes.^22–24^ In addition, because of tissue accumulation and residual sedation, it takes longer to perform a reliable NWT on midazolam sedation.^5,25^ Opioids are often used in combination with hypnotic agents to ensure analgesia and to reduce hypnotic dosage,^16,24^ disadvantages of opioids however include respiratory depression and withdrawal symptoms.^24^ In addition, both opioids as well as dexmedetomidine can cause clinically significant hypotension with reductions in mean arterial pressure accompanied by acute increases in ICP.^16,24,26–28^ Dexmedetomidine has gained interest for the potential benefits of reducing delirium without respiratory depressant effects.^24,29^ Additionally, ketamine can be used safely even in hemodynamically unstable TBI patients and without increases in ICP, and therefore, like etomidate, is used commonly as an induction agent.^5,15,30–32^ Barbiturates have long played an important role in sedation and managing alcohol withdrawal in TBI patients in the ICU.^33–35^ However, more recent studies highlighting elevated mortality with early barbiturate use^36^ have shifted practice and recommendations such that barbiturates are currently recommended for controlling elevated ICP refractory to maximum standard medical and surgical treatment.^6^ With both advantages and disadvantages of different sedative agents, no single sedative agent to date has shown to be more efficacious than the other leading to a wide variety in the use of sedative agents. However, adequately powered high-quality studies are lacking.

### Sedation Duration and NWTs

As shown in this study, the duration of sedation in TBI patients with increased ICP varied extensively between 24 h and 14 days. To date, it is not yet known what the most ideal sedation duration in this patient population is. Since sedative agents have many cerebral protective effects, the duration of sedation can play an important role in the prevention of secondary brain injury. Neurological wake-up tests (NWTs) are performed in 70.5% of patients but the frequency of these NWTs varied greatly in this study, consistent with pilot studies extracting this data directly from the electronic health record.^37^ While NWTs in sedated ICU patients are essential to perform a reliable neurologic exam,^38^ interruptions of sedation may induce increased cerebral metabolism and elevated blood pressure, both of which may result in brief increases in ICP and changes in cerebral perfusion pressure.^38^ High-quality studies are needed to investigate the ideal sedation duration in this patient population as well as the clinical benefits of NWTs.

### Sedation Depth

The Richmond Agitation Sedation Scale (RASS) can be used to assess sedation depth in TBI patients and to guide sedation therapy, although it may also be confounded in patients with motor injuries, critical illness myoneuropathy, or spinal cord injury. This study found great variation in RASS level in TBI patients in the ICU, highlighting that to date no studies have rigorously compared the ideal sedation depth in TBI patients in the ICU. It is known however that, in the general ICU population, moderate to deep sedation leads to prolonged mechanical ventilation and longer hospital length of stay.^39–41^ There is a need for further high-quality studies to investigate the ideal depth of sedation in TBI patients in the ICU, not only to encourage improved outcomes but also to prevent contamination of outcomes in randomized controlled trials. For example, the BEST:TRIP study concluded that TBI outcomes were not superior when care was based on ICP monitoring rather than imaging and clinical examinations, however, the protocol resulted in 24% receiving barbiturates in the ICP monitoring group and only 13% receiving barbiturates in the clinical monitoring group.^42^ Additionally, patients may have differential responses to the same sedation dose, and inadvertent burst-suppression documented on electroencephalography monitoring is common even in non-TBI ICU patients^43^ and mediates both delirium and mortality.^44,45^

To summarize, this study identified extensive practice-pattern variation in sedation management of neurotrauma patients in the ICU, whether due to a lack of evidence and institutional guidelines or due to patient variability related to pathoanatomic subtypes such as paroxysmal sympathetic hyperactivity occurring after diffuse axonal injury.^46^ It is unknown however if these differences also lead to different patient outcomes. The results of this study can be used for future comparative effectiveness research to investigate these specific differences to help optimize sedation strategies, develop institutional guidelines, and to promote patient outcomes.

### Strengths and Limitations

Acknowledging patient differences in response to sedation,^47^ this article represents the first internationally focused study examining practitioner differences in sedation strategies for critically ill TBI patients in the ICU. We acknowledge the limitations of survey-based investigations. Representative information was unavailable for many countries and the number of respondents per country was small. Additionally, this study did not well represent respondents working in middle- and low-income countries. Also, no detailed dosing approaches were surveyed. Lastly, because the survey was distributed through email, Twitter, and snowballing, we were unable to provide a response rate.

## Conclusions

There is great variability in the choice of sedative agents, duration of sedation, performance and frequency of NWTs and depth of sedation in TBI patients in the ICU, and institutional guidelines are uncommon. As a result, sedation strategies are chosen based on patient variation and practitioner preferences. Prospective comparative effectiveness studies investigating these specific sedation management differences are needed to help optimize sedation strategies, develop institutional guidelines, and promote patient outcomes.

## Supplemental Material

sj-docx-1-jic-10.1177_08850666231186563 - Supplemental material for Practice-Pattern Variation in Sedation of Neurotrauma Patients in the Intensive Care Unit: An International Survey Click here for additional data file. Supplemental material, sj-docx-1-jic-10.1177_08850666231186563 for Practice-Pattern Variation in Sedation of Neurotrauma Patients in the Intensive Care Unit: An International Survey by Rianne G.F. Dolmans, Brian V. Nahed, Faith C. Robertson, Wilco C. Peul, Eric S. Rosenthal and Marike L.D. Broekman in Journal of Intensive Care Medicine
