# Supplementary material for: Practice-Pattern Variation in Sedation of Neurotrauma Patients in the Intensive Care Unit: An International Survey
Source: J Intensive Care Med. 2023 Jul 7;38(12):1143–50. doi: 10.1177/08850666231186563 (PMC10616999; doi:10.1177/08850666231186563)
Supplement: sj-docx-1-jic-10.1177_08850666231186563 - Supplemental material for Practice-Pattern Variation in Sedation of Neurotrauma Patients in the Intensive Care Unit: An International Survey [file sj-docx-1-jic-10.1177_08850666231186563.docx]

**Supplementary Material: Survey**

| Informed consent | | |
| --- | --- | --- |
| Please review the linked consent information sheet.  I agree to participate in this research | | 0 Yes  0 No (if no, questionnaire completed) |
| Questions regarding sedation practices (the following questions relate to adults) | | |
| Do you treat adult patients with traumatic brain injury? | 0 Yes  0 No (if no, questionnaire completed) | |
| Do you routinely prescribe sedatives for procedural sedation, intubation, or ICU sedation? | 0 Yes  0 No (if no, questionnaire completed) | |
| Is there an institutional guideline on sedation practice for neurotrauma patients at your institution? | 0 Yes  0 No | |
| What induction sedative agents* do you use? [Multiselect]  ** Induction sedative agents (distinct from muscle relaxants) = used to safely facilitate endotracheal intubation in a manner that minimizes hemodynamic instability and secondary brain injury.*  *Please select all options that apply.* | 0 Propofol  0 Etomidate  0 Ketamine  0 Dexmedetomidine  0 Benzodiazepines  0 Opioids  0 Barbiturates  0 Antipsychotics  0 Clonidine  0 Other (please specify): …  0 Does not apply to me / I am not involved during the induction phase | |
| Why do you use these specific induction sedative agents? | 0 Institutional guidelines  0 Physicians preference  0 Other, please specify … | |
| What dose of induction sedative agents do you use?  *Please insert all sedatives + dosage* | [ Free text ] | |
| Do you use a uniform dose regimen for induction sedative agents in most neurotrauma patients? | 0 Yes  0 No | |
| What type of *maintenance* sedation medication* do you use?  [Multiselect]  ** Maintenance of sedation = as part of the overall management of TBI to permit mechanical ventilation and optimization of intracranial physiology.*  *Please select all options that apply.* | 0 Propofol  0 Etomidate  0 Ketamine  0 Dexmedetomidine  0 Benzodiazepines  0 Opioids  0 Barbiturates  0 Antipsychotics  0 Clonidine  0 Other (please specify): … | |
| Why do you use this certain sedation  *maintenance* medication? | 0 Institutional guidelines  0 Physicians preference  0 Other, please specify … | |
| Which dose of sedation *maintenance*  medication do you use?  *Please insert all sedatives + dosage* | [ Free text ] | |
| Do you use a uniform *maintenance* dose  regimen for all neurotrauma patients? | 0 Yes  0 No | |
| Do you routinely seek to change sedatives after the initial 24-48h? | 0 Yes  0 No | |
| Do you routinely use muscle relaxants during the maintenance phase in neurotrauma patients? [Branching question] | 0 Yes  0 No | |
| If yes, which muscle relaxants do you use? | 0 Rocuronium  0 Succinylcholine  0 Other | |
| What do you use as initial pharmacotherapy for elevated ICP? [Branching question] | 0 Osmotherapy  0 Sedation  0 Osmotherapy and sedation | |
| If osmotherapy, which osmotherapy do  you prefer as first line? | 0 Mannitol  0 Hypertonic saline | |
| If sedation, which sedative do you prefer  as first line therapy for elevated ICP? | 0 Propofol  0 Etomidate  0 Ketamine  0 Dexmedetomidine  0 Benzodiazepines  0 Opioids  0 Barbiturates  0 Antipsychotics  0 Clonidine  0 Other (please specify): … | |
| Sedation duration | | |
| Do you initially use a fixed duration of sedation or is sedation duration dependent on clinical condition? [Branching question] | 0 Fixed duration  0 Depends on clinical condition | |
| If you use a fixed duration of sedation, how  long do you keep neurotrauma patients  sedated? | [ Number of days ] | |
| If the sedation duration depends on clinical  condition, which clinical aspects are  evaluated? [Multiselect] | 0 Abnormal ICP or multimodality monitoring  0 Refractory seizures  0 Status Epilepticus  0 Paroxysmal Sympathetic Hyperactivity  0 RASS  0 Other, please specify … | |
| How long do you keep neurotrauma  patients with high ICP (ICP >20mmHg)  sedated? | [ Number of days ] | |
| How long do you keep neurotrauma  patients with refractory seizures or status  epilepticus sedated? | [ Number of days ] | |
| How long do you keep neurotrauma  patients with Paroxysmal Sympathetic  Hyperactivity sedated? | [ Number of days ] | |
| What RASS level are you aiming for in neurotrauma patients? | 0 +4 Combative  0 +3 Very agitated  0 +2 Agitated  0 +1 Restless  0 0 Alert and Calm  0 -1 Drowsy  0 -2 Light sedation  0 -3 Moderate sedation  0 -4 Deep sedation  0 -5 Unarousable | |
| Is a tracheostomy performed early in neurotrauma patients to minimize sedation? | 0 Yes  0 No | |
| Do you use sedation free intervals to do a  neurologic exam? [Branching question] | 0 Yes  0 No | |
| If yes, how often do you stop the sedation?  (hours between exams) | [ Number of hours ] | |
| How many minutes do you typically wait  to perform a neurological exam off  sedation? | [ Number in minutes ] | |
| What are indications for no sedation  interruption at your center or in your  practice? [Multiselect] | 0 ICP elevation  0 Herniation  0 Mass lesion  0 Seizures  0 Status epilepticus  0 Brain tissue hypoxia  0 Other, please specify … | |
| Side effects of sedation | | |
| Which side effects related to sedation medication do you encounter in patients with TBI in the ICU? | | |
| Hemodynamic instability (hypotension, bradycardia) | Never (0%) 1 2 3 4 5 6 7 8 9 10 Always (100%) | |
| Delirium | Never (0%) 1 2 3 4 5 6 7 8 9 10 Always (100%) | |
| Respiratory suppression | Never (0%) 1 2 3 4 5 6 7 8 9 10 Always (100%) | |
| Propofol infusion syndrome | Never (0%) 1 2 3 4 5 6 7 8 9 10 Always (100%) | |
| Adrenal suppression | Never (0%) 1 2 3 4 5 6 7 8 9 10 Always (100%) | |
| General questions | | |
| In which country do you practice? | [ List of countries ] | |
| Which best describes your institution? | 0 University Hospital/Academic Medical Center  0 Hospital with no academic affiliation  0 Community Hospital  0 Private Practice | |
| Which of the following best describes you? | 0 Resident  0 Fellow  0 Attending physician  0 Critical care nurse practitioner  0 Critical care physician assistant  0 Other | |
| Years of experience after terminal clinical training (residency/fellowship) | [Number in years] | |
| In what field did you complete/are you completing your residency? [Multiselect] | 0 Anesthesiology  0 Intensive Care  0 Neurosurgery  0 Emergency Medicine  0 Neurology  0 Other (please specify): … | |
| I deliver ICU care primarily: | 0 In a medical ICU  0 In a neuro ICU  0 In a surgical ICU  0 In a mixed ICU, predominantly neuro  0 In a mixed ICU, predominantly surgical  0 In a mixed ICU, predominantly medical  0 As a consultant to multiple ICUs  0 Other | |
| Additional comments | | |
| Please submit any additional comments here | [Free text] | |
